# Understanding the Production of Population Health and the Role of Paying for Population Health

**Published:** 2010-08-15

**Authors:** John Mullahy

**Affiliations:** Population Health Sciences, University of Wisconsin–Madison

## Abstract

This article considers 2 related themes that address population health outcomes and the contributions to those outcomes by time, place, individual behaviors and choices, and activities of various social sectors. First, what does it mean to "produce" population health, and how can the production of health be understood empirically? Second, through what processes can incentives be modified to improve population health? Among the issues that arise are understanding the mechanisms through which paying for population health works and how the health-producing incentives materialize in various sectors, especially those whose primary functions are not generally viewed as fostering better population health.

## Overview

Population health refers to the distribution of some measure of health status across members of a defined population at a defined point in time or during a specified time. Specific attributes of population health — its mean, its across-individual variation, and other attributes — are all properties of this underlying distribution of health in a population. Of concern here are several questions: How do the population health outcomes observed at any place and point in time arise? How and why do they vary over time and geography? What contributions to population health outcomes are made by people's behaviors and choices? And how do the activities of various sectors (producers and other institutions, broadly defined) ultimately influence population health outcomes?

I consider 2 related themes that address these questions. First, what does it mean to "produce" population health, and how can the production of health be understood empirically? The basic premise is that people respond to a variety of incentives and constraints ("opportunities") to make choices that promote or destroy their health. Second, because people respond to incentives to engage in health-enhancing activities, through what processes can incentives be modified to improve population health?

The central issues are understanding the mechanisms through which paying for population health (PPH) works and how health-producing incentives materialize in various sectors, especially those whose primary functions are not generally viewed as fostering better population health. How multiple sectors affect the health of populations and how incentives — financial, regulatory, cultural, psychological, or others — play a central role in the process is our main concern.

## Production of Health and Population Health

### The Grossman model of health production

Although alternative complementary and competing paradigms have been developed, the model developed by Michael Grossman in the early 1970s provides a basis for economic analysis of how health outcomes arise (1,2). Grossman's model was based on earlier work on general household production by Becker (3). In a simple version of the Grossman model, people are viewed as producers of health by the choices they make about their behaviors and their use of medical care. As an outcome of this process, lifetimes are more or less "healthy." People are constrained in their opportunities to produce health for various reasons: financial constraints, time constraints, baseline endowments of physical and mental health (known as health capital in the Grossman model), and the social and natural environments and contexts they occupy. Moreover, people may differentially weigh being healthy relative to other things and, consequently, may be more or less motivated to invest in their health even if confronted with the same opportunities to produce health.

The analytical framework that arises from this paradigm involves 2 main concepts: health production functions and choice or demand functions:

Healthiness = f_1_(x)

and

Choices = f_2_(p)

where x represents choices, health capital, and social and environmental factors, and p represents constraints and incentives, health capital, preferences, and social and environmental factors. The literature refers to h = f_1_(x) as the "structural" health production function and to h = f_3_(p) as the "reduced form" health production function. Therefore, h = f_3_(p) indirectly. Determining the shape of health production functions is ultimately an empiric matter.

Within a population, people generally differ in the constraints and incentives ("opportunity sets") they face (except that everyone has 1,440 minutes of time to spend each day), in the baseline levels of health capital possessed, in the values placed on healthiness versus other desirable ends, and in their capabilities to produce health via particular choices. This framework also emphasizes that the heterogeneous environments (eg, social, natural, cultural) that people occupy may influence health directly, as well as influence the choices that are made. Because of all these differences among people, the Grossman model inherently predicts that the healthiness within the population will vary and, thus, likely differ across populations. Virtually all of the empiric work that has been pursued in this field has been based on some variant of the Grossman model's conceptual framework.

Given the scope of this essay, it is not possible to elaborate on all the aspects of this paradigm. Three features, however, are noteworthy. First, the use of medical care is just one of many choices people make to invest in their health; to varying degrees, constraints such as health insurance, genetic predispositions, the value of time, and other factors affect choices made to use medical care. Debates about the productivity of medical care can in principle be undertaken in this conceptual framework. Second, the role of schooling in this framework has been debated extensively; better schooling correlates positively and strongly with better health, but the extent to which schooling (however measured) causes better health outcomes is not well understood (4). Third, health status is multidimensional, and various choices may differentially influence different aspects of health; some choices (eg, exercise) may influence positively a range of aspects of health, while others (eg, prescription drug use) may contribute positively to some aspects but negatively to others (eg, via adverse side effects).

### Empiric considerations

To be useful in forming policies and interventions, the Grossman model's conceptual framework must be supported by data. Unfortunately, there are nontrivial empiric impediments to understanding the parameters of the Grossman model, which is a reason why the health production function has been termed a fantasy equation (5). Significant progress has been made in the empiric understanding of health production relationships dating back to the first serious empiric work in the field (6); however, the limitations of using this analytical framework are as notable as the successes. Some of these empiric impediments include the following:

1. The availability of individual-level data on health, health-producing behaviors, and related phenomena has grown substantially in the 40 years since Grossman's original work was undertaken. However, the utility of such data has not grown commensurately. Whether the data are from government records or from health care administrative records, confidentiality regulations or other logistical issues often preclude linking these data to information on constraints, incentives, and environments faced by people. Without such information, the statistical obstacles to understanding relationships that produce population health will be formidable.
2. Data on aggregates of people are less constrained by confidentiality considerations, but there are limits on what can be learned from studying data obtained at aggregate levels because of ecologic fallacy (drawing inferences about people on the basis of empiric analysis that uses aggregate data) and related considerations. There is considerable population heterogeneity in the opportunity sets that people face, the social and environmental contexts that they occupy, and their preferences for health and other valued outcomes. Relying on aggregates of data (eg, geographic aggregates like census tracts or counties) could obscure potentially important within-aggregate heterogeneity.
3. Although considerable information is available on the health-related choices made by people, the Grossman model forces the recognition that such behaviors are self-selected (eg, because of differences in preferences across a population) rather than exogenously or randomly assigned. Consequently, simple regressions of health outcomes on health-related choices do not reveal the causal mechanisms fundamental to the Grossman framework. To circumvent such considerations of self-selection, it may be more instructive in some cases for policy making to directly relate health outcomes and opportunity sets (eg, by estimating the reduced-form health production function described above), thus avoiding or mitigating some of the concerns about self-selection. For instance, although it may be interesting to understand the causal relationship between milk consumption and various health outcomes, it might also be interesting to know — and may be easier to learn — whether dairy support or policies for vitamin D fortification affect health, albeit indirectly. Yet the obstacles identified above often preclude the linkage of suitable data on the opportunity set measures to personal data on health outcomes and other characteristics.
4. Even if these issues were resolved, there remain fundamental measurement issues. One issue is to reconcile the ideal conceptual measures of personal health with those available in our data. Another is how to summarize a heterogeneous distribution of health in a population in order to quantify "population health" in any particular instance.

## Producers and Institutions in the Production of Population Health

### Incentives and the production of health across sectors

The production of population health arises from the activities of population members producing health at the personal level, albeit influenced by and involved in social and natural environments in which such productive behaviors may be undertaken and interact socially. In this paradigm, "sectors" only "produce" health to the extent that their actions shape a person's social and environmental contexts: "sectors" don't produce health, people do.

If this personal health production paradigm is accepted, then what specific empiric meaning should be given to the notion of the "multisector production of health"? Without abandoning this term — it is useful to the extent that it has served to draw attention away from the health care sector, per se, as the "sector" from which population health outcomes arise — what specific interpretation can be given that is consistent with the personal-based model of the production of health? Instead of conceiving sectors as "producers" of population health outcomes, these different sectors and their activities ought to be viewed as promoters or inhibitors of improved population health outcomes. This seems reasonable to the extent that the policies and activities in which they engage establish incentives or disincentives that influence a person's choice of health-producing inputs (ie, affect a person's time, money, and other factors that define their opportunity sets). Consequently, PPH means deploying resources to create incentives. In essence, this framework provides economic and social actors with self-serving reasons to change the way business is done so that those clients affected within their spheres of activity might come to face new and (presumably) stronger incentives to make healthier choices.

The textbook version of this approach is an economy that produces "guns and butter" and that is constrained in the quantities of these commodities that can be produced by the quantities of resources (eg, labor, materials) available at any time in that economy. In this instance, however, "health" and "X" substitute for guns and butter as the 2 outputs of our sector, and the "health" outputs arise indirectly via the health-producing activities of the clients of this sector. For example, the activities of the K-12 education sector influence clients' health production activities but also affect, for example, their math and reading scores and college acceptance rates.

In this 2-commodity world, the mix of "health" and "X" that actually emerges is dictated by the incentives producers have to channel productive resources into these activities. In general, market or political forces or both will determine these incentives, but we recognize that policy interventions such as PPH can modify the nature and magnitude of the incentives that would be determined by market forces on their own (eg, agricultural policy, in the form of higher price subsidies or supports, would be expected to sway the production balance more toward butter). For instance, in the absence of a PPH strategy, the sector in question may have little or no incentive to provide incentives for its clients to produce health. A successful PPH strategy modifies the incentives facing this sector, resulting in an outcome that implies more "health" at the necessary cost of less "X." The practical issue is how incentives can be most cost-effectively provided to the various sectors, so that those sectors in turn provide incentives to their respective clients.

### Examples

Consider the range of activities in which K-12 policy makers engage that have potential implications for the health-producing activities of their clients. Considering the budgetary constraints and regulatory environments they face, K-12 school boards and administrators enact policies to achieve outcomes across a range of objectives (eg, test scores, dropout rates, vending machine and cafeteria offerings, foreign language classes, athletic team performance). The activities that contribute to these outcomes entail various incentives and disincentives for students to make health-producing choices. In the PPH context, paying for better population health outcomes via activity in the K-12 sector entails using financial and political muscle to change the importance that school boards and administrators attach to the outcomes they produce (eg, more focus on physical education, less on after-school clubs) and the regulatory environments in which they operate (eg, bans on soft drink machines, sponsorships). Over time the investments in after-school clubs could culminate in enhanced levels of human capital of the participants which in turn could enhance health-producing activities in the adult years.

These same principles apply in more specific contexts, and are illuminating when considering how various "sectors" of the economy (private and public) may be provided incentives via PPH strategies to engage in activities that result in incentives for people to engage in healthier behaviors. Transportation departments can invest more in highway beautification or in road safety; environmental and natural resource agencies can invest more in protecting endangered species or in preventing contamination of air and water; businesses can invest more in decorative art or in workplace wellness and fitness programs. The motivations for such differential investment strategies arise from the manner in which the various PPH strategies are implemented.

Two considerations are noteworthy. First, there are tradeoffs because of the ultimate scarcity of productive resources, at least in the short run; more production of health comes at a cost of less production of "X." Second, the mix of "health" and "X" produced does not arise randomly but rather by purposive choices made in the existing incentive structures by the people who are the clients of the sectors in question. Understanding how PPH strategies can modify existing incentive structures that are established implicitly or explicitly by the respective sectors — as well as the social and economic contexts that influence health that are under the purview of these sectors — is essential if the PPH approach is to be successful.

## Summary

Incentives matter. People make health-producing choices in light of the opportunity sets they face. Public and private sector institutions directly or indirectly establish incentives for people to be more or less healthy because of the incentives they themselves face. Consequently, PPH ultimately implies that financial, political, regulatory, and other resources must be deployed strategically to change the incentive structures to which people and institutions respond in their day-to-day activities. Considerations of the practicalities of how such incentive structures can be modified — and recognition of the tradeoffs that may be entailed in effecting such modifications — ought to be near or at the top of the PPH research agenda.

## Acknowledgments

This manuscript was developed as part of the Mobilizing Action Toward Community Health (MATCH) project funded by the Robert Wood Johnson Foundation. Thanks to Dave Kindig and Stephanie Robert for insightful comments on an earlier version of this essay. Partial financial support was provided by the Health and Society Scholars Program at the University of Wisconsin-Madison.
